# The [4Fe-4S] Cluster of HydF Is Essential for [FeFe]-Hydrogenase Maturation

**DOI:** 10.1021/jacs.5c18286

**Published:** 2025-12-10

**Authors:** Batuhan Balci, Eric M. Shepard, Alexander Marlott, Roark D. O’Neill, Michael T. Mock, William E. Broderick, Joan B. Broderick

**Affiliations:** Department of Chemistry & Biochemistry, 33052 Montana State University, Bozeman, Montana 59717, United States

## Abstract

The organometallic H-cluster of the [FeFe]-hydrogenase is assembled *in vivo* through a complex process requiring the action of three dedicated maturation enzymes, HydG, HydE, and HydF, as well as the aminomethyl-lipoyl-H-protein (H_met_) of the glycine cleavage system (GCS). Here we probe the role of HydF and its [4Fe-4S] cluster in [FeFe]-hydrogenase maturation by using a defined semisynthetic approach in which [Fe^I^_2_(μ-SH)_2_(CO)_4_(CN)_2_]^2–^ ([2Fe]_E_) is used to bypass HydE and HydG, and GCS components are used in place of cell lysate. We show that inclusion of the iron–sulfur carrier protein NfuA and the high-CO-affinity myoglobin variant Mb^H64L^ provides dramatically improved hydrogenase activities up to 828 μmol/min/mg, equivalent to the best reported activities for *Chlamydomonas reinhardtii* [FeFe]-hydrogenase isolated from the native organism. Apo-HydF lacking a [4Fe-4S] cluster provides very little hydrogenase activity; however, full maturation is restored with the addition of NfuA, which we demonstrate reconstitutes the [4Fe-4S] cluster of HydF. In addition, a HydF variant lacking a [4Fe-4S] cluster by changing two cysteine ligands to alanine is completely unable to support either semisynthetic maturation using [2Fe]_E_, or full maturation using HydG and HydE, even in the presence of NfuA, demonstrating that the HydF [4Fe-4S] cluster is absolutely essential for [FeFe]-hydrogenase maturation. The possibility that the HydF [4Fe-4S] cluster plays a role in direct binding of [2Fe]_E_ is negated by our results with the HydF^D311C^ variant, which demonstrate that the labile Asp311 cluster ligand is not essential for [2Fe]_E_ binding and HydA maturation. We therefore conclude that [2Fe]_E_ binds HydF adjacent to, but not directly coordinated to, the [4Fe-4S] cluster. The HydF [4Fe-4S] cluster is proposed to be essential due to its impact on the [2Fe]_E_ binding orientation and the ability of the HydF/[2Fe]_E_ complex to form productive interactions with H_met_ or the H_met_/T-protein complex during DTMA ligand biosynthesis.

## Graphical abstract

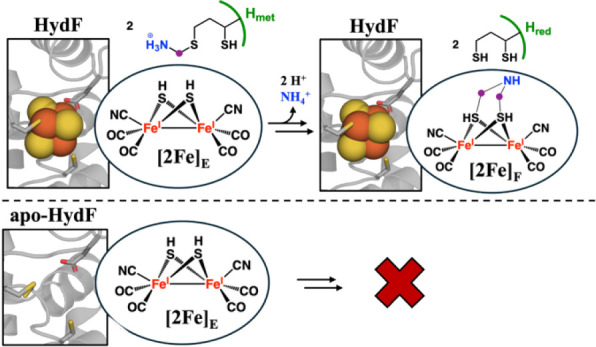

## Introduction

[FeFe]-hydrogenases harbor a biologically unique organometallic cofactor, the H-cluster, which is the site of catalysis for the reversible interconversion of protons and hydrogen, H_2_.^1–4^ The H-cluster consists of a [4Fe-4S] cluster ([4Fe-4S]_H_) coordinated by four conserved cysteine residues and bridged to a [2Fe] organometallic subcluster ([2Fe]_H_) coordinated by three carbon monoxide (CO) and two cyanide (CN^–^) ligands as well as a bridging dithiomethylamine (DTMA, Figure 1).^5–8^ While the [4Fe-4S]_H_ is built by housekeeping iron–sulfur cluster assembly machinery,^9–13^ the synthesis and installation of the [2Fe]_H_ is a distinct process requiring dedicated [FeFe]-hydrogenase maturation enzymes.^14–17^

**Figure 1:**
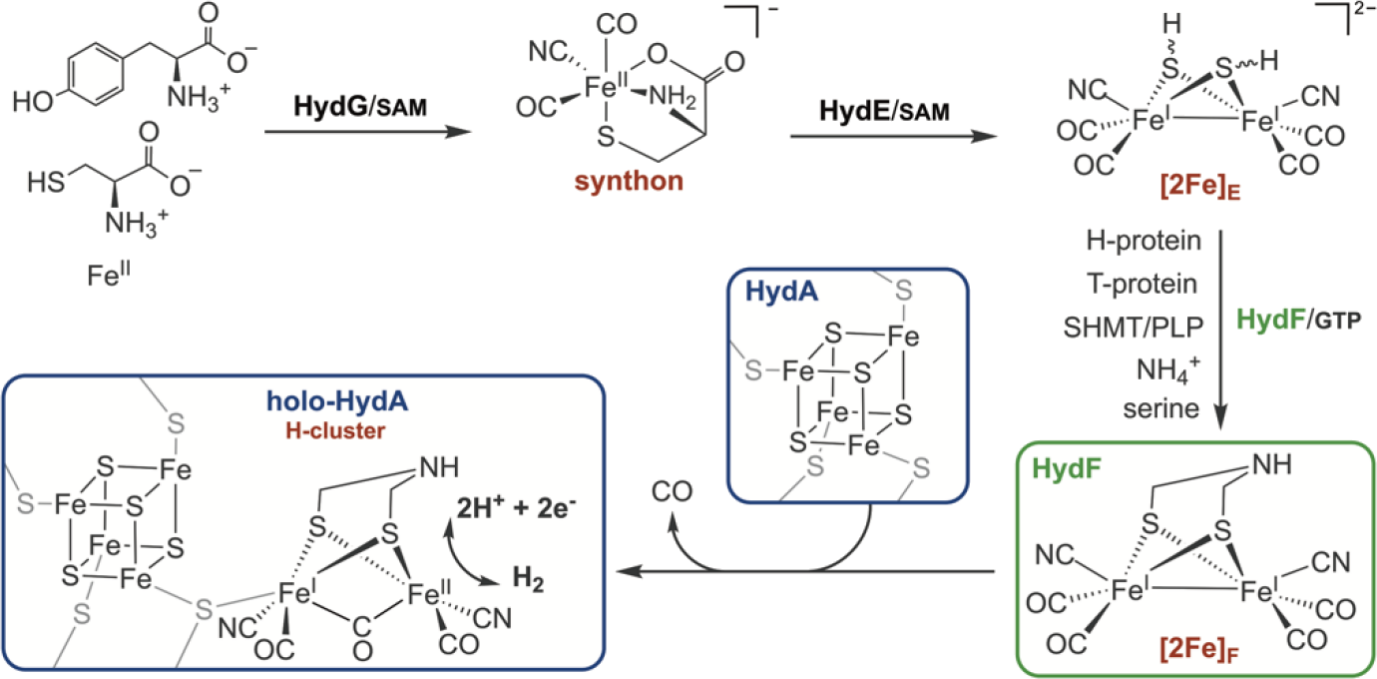
Maturation of the [FeFe]-hydrogenase. The radical SAM enzyme HydG synthesizes the CO and CN^–^ ligated synthon, which is then reduced and dimerized to form [2Fe]_E_ by the radical SAM enzyme HydE. The dithiomethylamine ligand is synthesized on [2Fe]_E_ bound to HydF, via the aminomethyl-lipoyl H-protein. The [2Fe]_F_ cluster is transferred to HydA to make the intact 6Fe H-cluster of holo-HydA.

[FeFe]-hydrogenase maturation starts with HydG, a radical *S*-adenosyl-l-methionine (SAM) enzyme that cleaves tyrosine at an N-terminal radical SAM [4Fe-4S] cluster to generate the diatomic ligands CO and CN^–^.^18,19^ The CO and CN^–^ coordinate the fifth iron of the C-terminal [4Fe-4S]-Fe­(Cys) cluster, with two tyrosine turnovers leading to formation of a mononuclear [Fe^II^(κ^3^-cys)­(CN)­(CO)_2_]^−^ synthon (Figure 1).^19–26^ The mononuclear Fe^II^ synthon has been shown to be directly transferred from HydG to the radical SAM maturase HydE.^27^ Spectroscopic and crystallographic studies using the synthetic analog of the synthon, syn-B, have shown that the synthon binds to HydE, and HydE reduces it via a radical-addition adenosylation reaction to generate an Fe^I^ species as detected by EPR spectroscopy.^28,29^ These findings led to the hypothesis that HydE ultimately catalyzes the formation of a Fe­(I)­Fe­(I) dimeric product [Fe^I^_2_(μ-SH)_2_(CO)_4_(CN)_2_]^2–^ ([2Fe]_E_, Figure 1).^17^ Synthesis of [2Fe]_E_ enabled semisynthetic maturation, wherein [2Fe]_E_ replaced the enzymes HydG and HydE, and in the presence of HydF, *E. coli* cell lysate, and apo-HydA, led to the formation of an active [FeFe]-hydrogenase, consistent with [2Fe]_E_ being the product of HydE catalysis and demonstrating that HydE is not responsible for DTMA biosynthesis.^30^ Recent work has provided direct spectroscopic evidence for the formation of [2Fe]_E_ on HydE.^31^

With HydE eliminated as the catalyst for DTMA biosynthesis,^30^ attention turned to HydF and/or components of the cell lysate, the latter of which was necessary for *in vitro* maturation but for unknown reasons. The mystery of DTMA biosynthesis was solved through the identification of the aminomethyl-lipoyl-H-protein (H_met_) of the glycine cleavage system (GCS) as the missing link and led to the development of a fully defined lysate-free *in vitro* [FeFe]-hydrogenase maturation system, wherein H_met_ was generated *in situ* via GCS components.^32^ DTMA is proposed to be synthesized on [2Fe]_E_ through the interaction of a HydF-[2Fe]_E_ complex with H_met_ to install the C–N–C backbone, which is supported by the results of a fully defined semisynthetic maturation reaction using [2Fe]_E_, HydF, and GCS components.^32,33^ The proposed mechanism for DTMA biosynthesis involves nucleophilic attack of the bridging sulfides of [2Fe]_E_ on the C of an aminomethyl group of H_met_, resulting in aminomethylation of the bridging sulfides of [2Fe]_E_ (Scheme 1).^32^ Condensation of the aminomethyl moieties with loss of NH_4_^+^ would then form the DTMA and the immediate H-cluster precursor [Fe^I^_2_(DTMA)­(CO)_4_(CN)_2_]^2–^, [2Fe]_F_ (Scheme 1).^32^ Rauchfuss and co-workers synthesized the proposed diaminomethylated-[2Fe]_E_ intermediate, and showed it was capable of maturing the [FeFe]-hydrogenase, providing support for this mechanism.^34^ The H_met_ central to DTMA ligand biosynthesis is formed by the T-protein of the GCS and SHMT, with serine and ammonium providing the C and N backbone atoms of DTMA.^32,35^

**Scheme 1:**
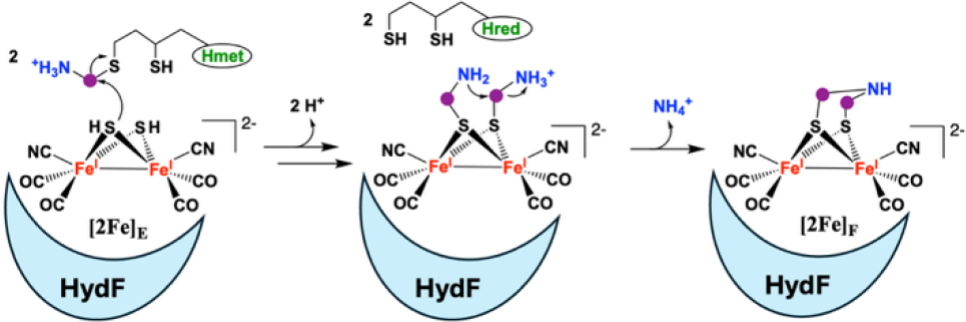
Synthesis of DTMA on the HydF-[2Fe]_E_ Complex

HydF plays a central role in the [FeFe]-hydrogenase maturation process, scaffolding the [2Fe] precursor for final processing before delivering it to the apo-HydA to form the catalytically active enzyme.^36–39^ A HydF-bound iron–sulfur cluster was implicated as important to maturation by early *in vivo* studies.^40^ While the [4Fe-4S] cluster of HydF has been extensively studied using spectroscopic approaches,^39,41–49^ and has been visualized in an X-ray crystal structure of the protein,^50^ the precise role of this cluster in maturation remains unclear. Synthesis of [2Fe]_F_ ^51^ allowed semisynthetic maturation studies, which demonstrated that HydF transfers [2Fe]_F_ to HydA, with loss of CO to yield active holo-HydA;^52,53^ spectroscopic evidence suggested that [2Fe]_F_ in these studies was coordinated to the HydF [4Fe-4S] cluster via a bridging cyanide.^52^ Biochemical loading of HydF using HydG and HydE led to a similar proposed cyanide bridge between [2Fe]_F_ and the [4Fe-4S] cluster.^48^ However, recent work by Happe and co-workers showed that the [4Fe-4S] cluster of HydF is not required for maturation of the [FeFe]-hydrogenase when using synthetic [2Fe]_F_.^54^ They further provided evidence for a conserved binding pocket for the [2Fe]_F_ cluster that is located in proximity to the [4Fe-4S] cluster.^55^ The apparent involvement of the HydF iron–sulfur cluster during *in vivo* maturation,^40^ but not for maturation with synthetic [2Fe]_F_,^54^ as well as the differing models for [2Fe]_F_ binding to HydF,^41,48,52,55^ raises important questions about the role of the HydF [4Fe-4S] cluster during maturation.

We report here the full optimization of our [2Fe]_E_-based defined semisynthetic [FeFe]-hydrogenase maturation system that yields fully mature [FeFe]-hydrogenase with specific activities comparable to those of the *in vivo* produced enzyme in as little as 2 h. This maturation system includes the use of the *E. coli* iron–sulfur carrier protein NfuA and a high-affinity CO-binding H64L myoglobin variant, as recently reported for the full enzymatic *in vitro* [FeFe]-hydrogenase maturation.^56^ We further use a combination of site-directed mutagenesis, spectroscopy, and our optimized [2Fe]_E_-based semisynthetic *in vitro* maturation system, as well as the full enzymatic maturation system, to probe the role of the [4Fe-4S] cluster of HydF in [FeFe]-hydrogenase maturation. Maturation reactions using apo-HydF (WT HydF lacking the [4Fe-4S] cluster) and those using a HydF cluster knockout variant demonstrate that the [4Fe-4S] cluster of HydF is absolutely essential for semisynthetic maturation with [2Fe]_E_, as well as for the full enzymatic maturation using HydG and HydE. However, the [4Fe-4S] cluster is not required for binding [2Fe]_E_ to HydF, with results suggesting that direct coordination of [2Fe]_E_ to HydF is not required for maturation. The implications of these results for the role of the HydF [4Fe-4S] cluster in the synthesis of the DTMA ligand during maturation of the [FeFe]-hydrogenase are discussed.

## Results

### Optimization of Defined Semisynthetic Maturation for High [FeFe]-Hydrogenase Activity

Previous reports on semisynthetic [FeFe]-hydrogenase maturations using synthetic [2Fe]_E_ to bypass HydG and HydE, carried out either in the presence of cell lysate or in a defined system, provided specific activities <100 μmol H_2_ min^–1^ mg^–1^ HydA, substantially lower than those observed for *Chlamydomonas reinhardtii* [FeFe]-hydrogenase (*Cr*HydA) matured *in vivo*.^30,33,57,58^ We have modified our previously reported^33^ defined [2Fe]_E_-based semisynthetic maturation reaction in order to exclude components that, in principle, should not be required due to bypassing HydG and HydE, such as the iron (as ferrous sulfate, FS) required for synthon formation by HydG. In all cases, a 10:1 ratio of HydF to HydA was used in these maturations, consistent with prior work^37,39,50,52,59^ and verified to be optimal for achieving high hydrogenase activities in the [2Fe]_E_-based semisynthetic maturations reported here (Figure S1). Maturations carried out using HydF, [2Fe]_E_, SHMT, PLP, T-protein, serine, ammonium, and HydA but without added FS provided an activity of 34 ± 17 μmol H_2_ min^–1^ mg^–1^ HydA after 4 h (Figure 2), which is approximately half the hydrogenase activity observed at 4 h in the original defined semisynthetic maturation that included FS, suggesting that iron plays a role even during [2Fe]_E_-based semisynthetic maturation.^33^

**Figure 2:**
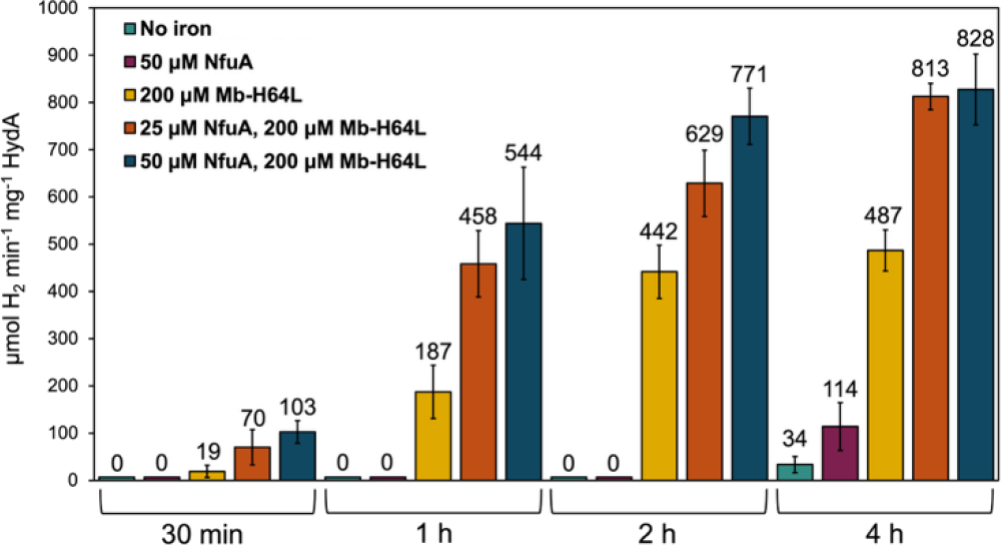
Semisynthetic maturation of *Cr*HydA using wild type *Ca*HydF and [2Fe]_E_ with or without Mb^H64L^ and NfuA. H_2_ production activity of each maturation reaction was measured at 30 min, 1, 2, and 4 h. Green, no NfuA or Mb^H64L^; red, 50 μM NfuA dimer; yellow, 200 μM Mb^H64L^; orange, 25 μM NfuA dimer and 200 μM Mb^H64L^; blue, 50 μM NfuA dimer and 200 μM Mb^H64L^.

Recent work has shown that the iron–sulfur carrier protein NfuA significantly improves the results of the defined enzymatic maturation using all three maturase enzymes, due to its ability to reconstitute iron–sulfur clusters and reload the “dangler” iron of HydG, where the synthon is built.^56^ The high-CO-affinity myoglobin H64L variant (Mb^H64L^) has also been shown to have a significant impact on hydrogenase activities during full enzymatic defined maturation reactions due to its ability to bind free CO and prevent formation of CO-inhibited [FeFe]-hydrogenase.^56^ To probe whether NfuA and Mb^H64L^ could have similar impacts on semisynthetic maturation reactions that bypass HydG and HydE, [FeFe]-hydrogenase maturation reactions using [2Fe]_E_ were carried out in the presence of NfuA, in the presence of Mb^H64L^, or in the presence of both proteins (Figure 2). Inclusion of 50 μM NfuA dimer harboring a [4Fe-4S] cluster in the defined semisynthetic maturation improved the hydrogen production activity at 4 h to 114 ± 51 μmol H_2_ min^–1^ mg^–1^ HydA, a nearly 4-fold increase in activity relative to maturation reactions with no added iron (Figure 2).

To probe whether sequestering free CO has an impact on [2Fe]_E_-based [FeFe]-hydrogenase maturation, we carried out maturation reactions in the presence of Mb^H64L^. Inclusion of 200 μM Mb^H64L^ in the maturation reaction in the absence of any other exogenous iron provided a dramatic enhancement in hydrogenase activity, with activities of 487 ± 43 μmol of H_2_ min^–1^ mg^–1^ HydA at 4 h (Figure 2). This is a substantial improvement in the semisynthetic maturation of HydA with [2Fe]_E_, with activities significantly surpassing values previously reported.^30,33,57^ These results support our hypothesis that the production of free CO appreciably limits hydrogenase activities achieved during semisynthetic maturation.

To investigate the combined effect of both NfuA and Mb^H64L^ during the [2Fe]_E_-based semisynthetic maturation, assays were carried out in the presence of 25 or 50 μM NfuA, in addition to 200 μM Mb^H64L^. Results demonstrated that the maturation assays provided fully matured *Cr*HydA with a specific activity of >800 μmol H_2_ min^–1^ mg^–1^ HydA within 4 h of incubation, a number comparable to that of *in vivo* matured *Cr*HydA (Figure 2).^30,33,57,58^ The combined addition of NfuA and Mb^H64L^ provides a strong synergistic effect on maturation, dramatically increasing specific activities relative to the addition of the individual components. A similar effect was also observed for the full *in vitro* maturation.^56^

### Involvement of the HydF [4Fe-4S] Cluster during Maturation

In order to probe the role of the HydF [4Fe-4S] cluster during maturation, we expressed and purified *Clostridium acetobutylicum* HydF (*Ca*HydF) in the apo form (Figure S2); apo-HydF had no detectable iron and no UV–visible features characteristic of a [4Fe-4S] cluster (Figure 3A). Semisynthetic [2Fe]_E_-based maturation assays carried out with apo-HydF in the absence of added FS or NfuA, but in the presence of 200 μM Mb^H64L^, provided very little [FeFe]-hydrogenase activity at 1 and 2 h (Figure 4), consistent with early reports that the [4Fe-4S] cluster of HydF is important for *in vivo* [FeFe]-hydrogenase maturation.^40^ The activity increased to 85 μmol of H_2_ min^–1^ mg^–1^ HydA at 4 h, which we attribute to mobilization of iron, presumably from HydA, to reconstitute a small amount of the HydF [4Fe-4S] cluster. Inclusion of 50 or 100 μM NfuA and 200 μM Mb^H64L^ in the semisynthetic *in vitro* maturation reactions with apo-HydF dramatically increased the resulting [FeFe]-hydrogenase activities to ∼700 μmol H_2_ min^–1^ mg^–1^ HydA at 4 h (Figure 4), consistent with the hypothesis that NfuA serves to reconstitute a functionally essential [4Fe-4S] cluster on HydF. We directly probed the ability of NfuA to reconstitute a [4Fe-4S] cluster on HydF by incubating apo-HydF with holo-NfuA for 60 min in the presence of reducing agent. Apo-HydF does not exhibit an EPR signal, while holo-NfuA shows only a weak axial [4Fe-4S]^+^ cluster EPR signal (Figure 5). The EPR spectrum for the sample after incubating apo-HydF with holo-NfuA is characteristic of the HydF [4Fe-4S]^+^ cluster (Figure 5 and Figure S3),^46,48^ demonstrating that NfuA has reconstituted apo-HydF to its holo form with an intact [4Fe-4S] cluster. This process of HydF cluster loading reflects what also occurs during the *in vitro* maturation reactions in the presence of NfuA, which are carried out under similar reducing conditions (Figure 4).

**Figure 3:**
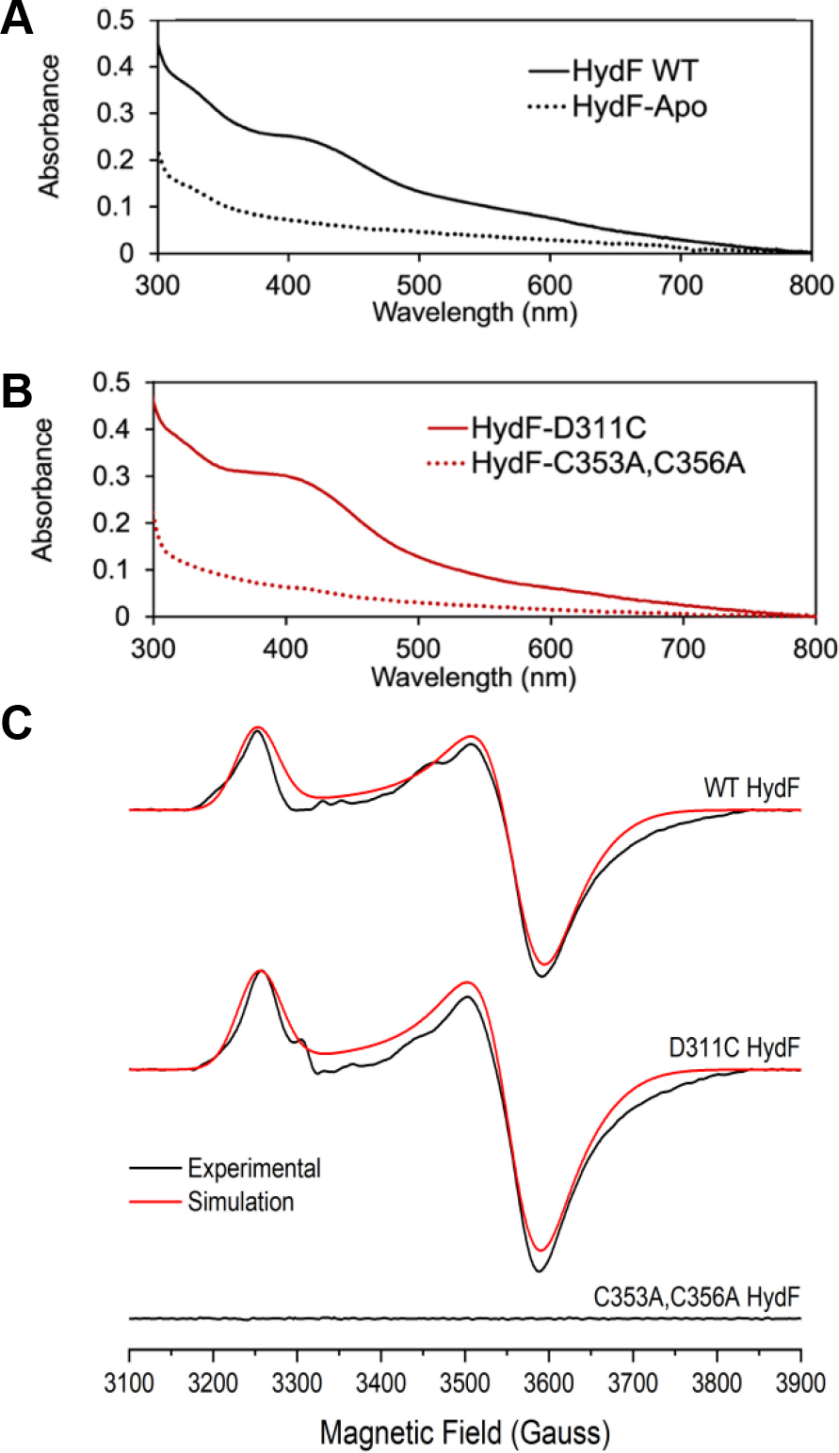
Spectroscopic characterization of HydF and its variants. (A) UV–vis spectra of WT and apo-HydF. (B) UV–vis spectra of HydF variants HydF^C353A,C356A^ and HydF^D311C^. (C) CW X-band EPR spectra (black) and simulations (red) for reduced WT HydF (45 μM, *g* = 2.058, 1.877, and 1.863), HydF^D311C^ (40 μM, *g* = 2.057, 1.880, and 1.863), and HydF^C353A,C356A^ (40 μM) in the presence of NaDT. EPR parameters: 12 K, 1 mW microwave power, 10 G modulation amplitude, and 100 Hz modulation frequency.

**Figure 4:**
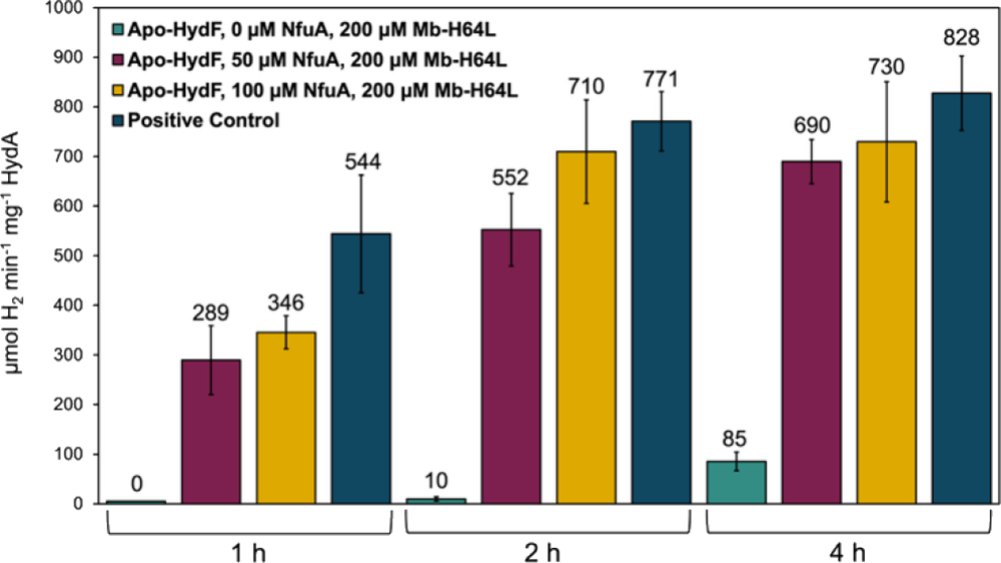
Maturation of *Cr*HydA using apo-*Ca*HydF with or without Mb^H64L^ and NfuA. H_2_ production activity of each maturation reaction was measured at 1, 2, and 4 h. Green, without NfuA but with 200 μM Mb^H64L^; purple, with 50 μM NfuA (dimer) and 200 μM Mb^H64L^; yellow, with 100 μM NfuA (dimer) and 200 μM Mb^H64L^; dark blue, positive control using WT *Ca*HydF with 50 μM NfuA (dimer) and 200 μM Mb^H64L^.

**Figure 5:**
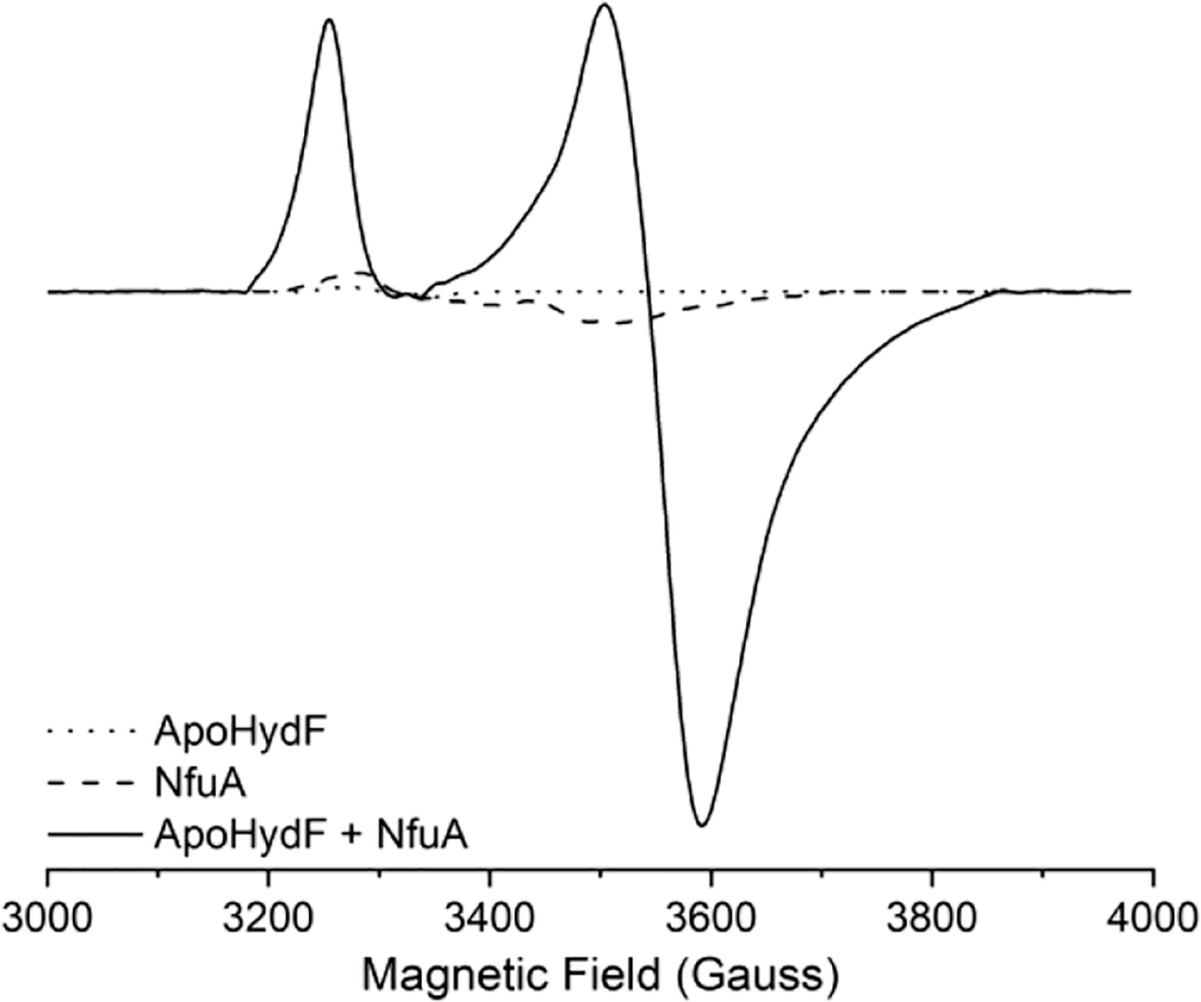
X-band CW EPR spectra demonstrating the loading of a [4Fe-4S]^+^ cluster on apo-HydF by holo-NfuA. Solid line, apoHydF (40 μM) incubated with 50 μM NfuA (dimer); dashed line, 50 μM NfuA (dimer); dotted line, apoHydF (40 μM). All samples were incubated for 1 h in the presence of 1 mM DTT and 2 mM NaDT. EPR parameters: 12.0 K, 1 mW microwave power, 10 G modulation amplitude and 100 kHz modulation frequency.

To further investigate the necessity of the [4Fe-4S] cluster of HydF during [FeFe]-hydrogenase maturation, we generated a HydF variant in which two of the conserved cluster-binding cysteines, C353 and C356, were changed to alanine residues. The resulting purified HydF^C353A,C356A^ variant had no detectable iron bound (even after attempted reconstitution with iron and sulfide), exhibited a UV–visible spectrum identical to that of apo-HydF, and was not EPR-active (Figure 3B,C), indicating the complete lack of an iron–sulfur cluster in this variant. Semisynthetic maturation assays using [2Fe]_E_ and the HydF^C353A,C356A^ variant in the presence of 50 μM NfuA and 200 μM Mb^H64L^ resulted in no detectable H_2_ production, reflecting no matured *Cr*HydA (Figure 6). To probe the involvement of the HydF [4Fe-4S] cluster in full enzymatic maturation reactions, we carried out defined maturation reactions using HydG, HydE, and HydF^C353A,C356A^, together with NfuA and Mb^H64L^; no H_2_ production was observed for the resulting *Cr*HydA (Figure S4). Together, these results demonstrate that the [4Fe-4S] cluster of HydF is absolutely essential for successful maturation via either the full enzymatic system or a semisynthetic route involving [2Fe]_E_.

**Figure 6:**
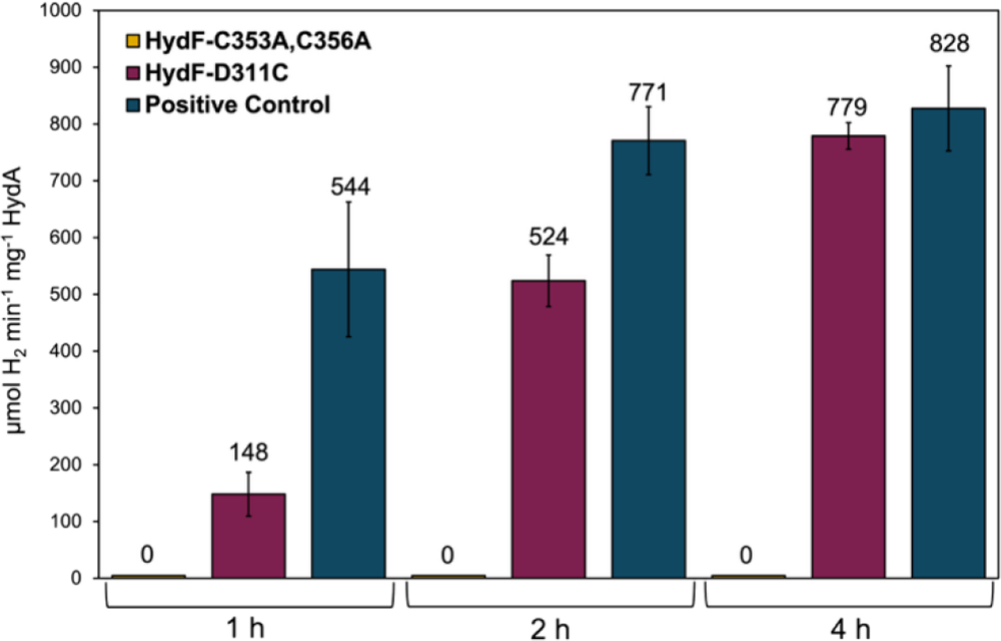
Hydrogenase activity at various incubation times after the maturation of *Cr*HydA using HydF variant proteins in the presence of 50 μM NfuA (dimer) and 200 μM Mb^H64L^. H_2_ production activity of each maturation reaction was measured at 1, 2, and 4 h for assays containing either HydF^C353A,C356A^ (yellow), HydF^D311C^ (purple), or WT (dark blue).

The HydF [4Fe-4S] cluster is coordinated by three conserved cysteine residues, with Glu or Asp providing the fourth ligand.^50^ This Asp/Glu ligand has been proposed to be labile, opening up a coordination site for a CN^–^ ligand of the [2Fe]_F_ cluster to bridge to the [4Fe-4S] cluster, an interaction that has been thought to be central to [2Fe] subcluster binding to HydF.^48,52^ In order to probe the importance of this conserved cluster ligand, we expressed, purified, and reconstituted the HydF^D311C^ variant; a cysteine ligand in this position should be less labile than an aspartate, and would not be expected to be displaced by a [2Fe]_E_ CN^–^ bridging ligand. The purified HydF^D311C^ binds a [4Fe-4S] cluster based on atomic absorption analysis as well as UV–visible and EPR spectroscopies, with the spectroscopic properties being essentially identical to those of the WT protein (Figure 3). Semisynthetic maturation assays with HydF^D311C^ and synthetic [2Fe]_E_ result in active [FeFe]-hydrogenase, with activities at 1 and 2 h being 148 and 524 μmol H_2_ min^–1^ mg^–1^ HydA, respectively, substantially below those for WT HydF at the same time intervals. However, at 4 h, the activity achieved with HydF^D311C^ (779 ± 23) is the same as that for WT HydF (828 ± 75), within error (Figure 6). These results indicate that the D311 ligand to the HydF [4Fe-4S] cluster is not essential for maturation and is consistent with the hypothesis that direct coordination of [2Fe]_E_ to the [4Fe-4S] cluster of HydF is not required in order to support maturation of apo-HydA.

### HydF [4Fe-4S] Cluster and Binding of [2Fe]_E_

Previously, we demonstrated via UV-visible, EPR, and atomic absorption (AA) spectroscopies that [2Fe]_E_ binds to WT, [4Fe-4S]–HydF.^33^ In order to probe [2Fe]_E_ binding to the HydF^D311C^ protein, 5 equiv of synthetic [2Fe]_E_ was added to the variant protein and allowed to incubate for 15 min, and then the sample was extensively buffer-exchanged. AA analysis demonstrates an increase in iron number from 3.92 ± 0.2 Fe/monomer to 5.39 ± 0.4 post-treatment, consistent with binding of [2Fe]_E_ to HydF^D311C^ despite the lack of a labile aspartate ligand on the [4Fe-4S] cluster. In order to determine whether the [4Fe-4S] cluster is required for the binding of [2Fe]_E_ to HydF, we used the same methodology to probe the binding of [2Fe]_E_ to both apo-HydF and HydF^C353A,C356A^ proteins. The results show an increase in the iron number from 0 to 1.80 ± 0.06 Fe/monomer for apo-HydF and from 0 to 1.75 ± 0.21 for HydF^C353A,C356A^, demonstrating that [2Fe]_E_ binds to HydF independent of the presence of the [4Fe-4S] cluster.

## Discussion

Biosynthesis of the organometallic H-cluster required for enzymatic activity of the [FeFe]-hydrogenase is a complex multistep process requiring multiple dedicated Hyd maturation enzymes, as well as other protein components and small molecule substrates, as shown in Figure 1.^15,32,56^ Use of synthetic compounds known or proposed to be products of each successive maturation enzyme, such as the synthon (HydG product), [2Fe]_E_ (HydE product), and [2Fe]_F_ (HydF product) (Figure 1), has provided the ability to bypass one or more maturation enzymes (semisynthetic approach) and thus simplified studies to focus on the functions of HydG, HydE, and HydF individually.^23,28–31,33,52,53,57,59–61^ HydF is a complex multifunctional maturation enzyme: it has been shown to have GTPase activity,^36,39,62^ to bind iron–sulfur clusters,^36,42–47,50,63,64^ to serve as a scaffold during H-cluster biogenesis,^37–39,41,48^ to transfer [2Fe]_F_ to apo-HydA to generate the enzymatically active holo-HydA,^37,39,52^ and most recently to play a role in catalyzing DTMA ligand biosynthesis (Scheme 1).^15,32,33^ Studies of the HydF iron–sulfur cluster during [FeFe]-hydrogenase maturation have led to differing conclusions regarding its involvement in the process.^40,54,59^ Here, we use our optimized [2Fe]_E_-based semisynthetic and full maturation reactions to provide new insights into the essentiality and role of the HydF [4Fe-4S] cluster during maturation.

The refinements to our [2Fe]_E_-based defined semisynthetic maturation system described herein have led to the ability to achieve full [FeFe]-hydrogenase activity comparable to that of the *in vivo* produced enzyme in the relatively short time frame of 2–4 h, providing us with a powerful platform to probe the role of the HydF [4Fe-4S] cluster. The keys to the improved maturation system included using the iron–sulfur carrier protein NfuA in place of FS, and the high-CO-affinity Mb^H64L^ variant to sequester free CO produced during maturation due to [2Fe]_E_ decomposition as well as CO loss during transfer of [2Fe]_F_ from HydF to HydA (see Figure 1). Sequestering CO prevents it from binding to the mature H-cluster to generate the catalytically inactive CO-inhibited state of the [FeFe]-hydrogenase.^7,65^

The essentiality of the HydF [4Fe-4S] cluster in [FeFe]-hydrogenase maturation was probed via the use of both apo-HydF and the HydF^C353A,C356A^ cluster knockout in our [2Fe]_E_-based defined semisynthetic maturation. Maturation reactions with apo-HydF provided very little hydrogenase activity, pointing to a critical role for the HydF [4Fe-4S] cluster. However, addition of NfuA to the maturation restored the ability to produce fully active HydA, consistent with NfuA acting to reconstitute the [4Fe-4S] cluster of HydF. EPR spectroscopy provided direct evidence for NfuA functioning to reconstitute the [4Fe-4S] cluster of apo-HydF (Figures 4 and 5). Use of the HydF^C353A,C356A^ cluster knockout variant provided a more rigorous test of the essentiality of the [4Fe-4S] cluster of HydF in maturation; this variant was incapable of supporting [2Fe]_E_-based semisynthetic maturation, even in the presence of NfuA and Mb^H64L^. The HydF cluster knockout variant was also completely incapable of supporting maturation in full *in vitro* maturation reactions employing HydE and HydG rather than [2Fe]_E_ (Figure S4). Together, these results unequivocally demonstrate that the [4Fe-4S] cluster of HydF is absolutely essential for *in vitro* [FeFe]-hydrogenase maturation.

Possible roles for the HydF [4Fe-4S] cluster could include binding the CO released during transfer of [2Fe]_F_ to HydA (Figure 1) or facilitating the redox chemistry involved in going from the Fe^I^–Fe^I^ [2Fe]_F_ species to the Fe^II^–Fe^I^ H-cluster, as has been previously proposed.^53^ However, Happe and co-workers recently reported that the HydF [4Fe-4S] cluster is *not* required for HydF to bind to synthetic [2Fe]_F_ (referred to as [2Fe]^MIM^ in their paper) and transfer it to HydA to form an active hydrogenase.^54^ This is surprising given the results presented here and the early *in vivo* evidence from King and Posewitz for an essential iron–sulfur cluster on HydF.^40^ Resolving these seemingly contradictory results requires invoking a crucial role for the HydF [4Fe-4S] cluster in a step prior to [2Fe]_F_ transfer to HydA. Given that DTMA ligand biosynthesis to form [2Fe]_F_ occurs via the interaction of H_met_ with the HydF/[2Fe]_E_ complex (Scheme 1), we propose that the HydF [4Fe-4S] cluster plays an essential role during the installation of the DTMA ligand on [2Fe]_E_ to form [2Fe]_F_. Among the possibilities, the HydF [4Fe-4S] cluster could function (i) to structurally create a proper binding site for [2Fe]_E_; (ii) to directly coordinate [2Fe]_E_ via a bridging CN^–^ ligand as previously proposed for [2Fe]_F_;^48,52^ or (iii) to play a role in modulating protein–protein interactions between HydF/[2Fe]_E_ and H_met_ (Scheme 1).

Regarding [2Fe]_E_ binding, the results presented here demonstrate that synthetic [2Fe]_E_ binds to HydF independent of the presence of the [4Fe-4S] cluster. However, the HydF/[2Fe]_E_ complex is competent for [FeFe]-hydrogenase maturation only when the [4Fe-4S] cluster is present. The possibility of direct binding of [2Fe]_E_ to the HydF [4Fe-4S] cluster is refuted by our results with the HydF^D311C^ variant, where the labile Asp311 cluster ligand is changed to a cysteine. This HydF^D311C^ variant harbors a [4Fe-4S] cluster; however, despite lacking the labile Asp ligand, this variant still binds [2Fe]_E_ and supports subsequent HydA maturation. We therefore conclude that [2Fe]_E_, like the [2Fe]_F_ studied by Happe and co-workers,^54,55^ binds in an adjacent site on HydF without direct coordination to the [4Fe-4S] cluster. This is consistent with prior results in which binding of [2Fe]_E_ to HydF caused an increase in EPR signal intensity of the HydF [4Fe-4S]^+^ cluster, but without line shape perturbations, as might be expected for [2Fe]_E_ binding near the [4Fe-4S] cluster, affecting its relaxation rate, but not binding directly to the cluster.^33^ The HydF [4Fe-4S] cluster could, however, impact the orientation and environment of the bound [2Fe]_E_. Consistent with this idea, Haas et al. predicted via docking studies that [2Fe]_F_ can bind in various orientations in the cavity adjacent to the [4Fe-4S] cluster,^55^ a prediction reproduced similarly in our own docking studies with [2Fe]_E_ (Figure S5). Further, HydF has been shown to undergo substantial local structural perturbations when the [4Fe-4S] cluster is bound,^50^ indicating that [4Fe-4S] cluster binding could impact both [2Fe]_E_ binding orientation and the ability of HydF/[2Fe]_E_ to form productive interactions with H_met_ or the H_met_/T-protein complex during DTMA biosynthesis. These interactions could be further influenced by GTP binding and hydrolysis, as the HydF GTPase domain has been implicated as a molecular switch.^62^

## Conclusions

The work presented herein uses an optimized *in vitro* maturation system to provide a clear demonstration that the [4Fe-4S] cluster of HydF is essential to [FeFe]-hydrogenase maturation. Further, our results using the [2Fe]_E_ precursor localize the crucial role of the HydF [4Fe-4S] cluster to processes occurring during the conversion of [2Fe]_E_ to [2Fe]_F_ on HydF. While the precise function of the HydF [4Fe-4S] cluster awaits further studies, possibilities include influencing productive binding of [2Fe]_E_, and/or facilitating interactions of HydF with H_met_ for DTMA ligand biosynthesis.

## Materials and Methods

### General Methods

Overexpression of all the target genes was done aerobically. Cell lysis and purification of target-tagged proteins were conducted under anaerobic conditions in a N_2_/H_2_ atmosphere Coy chamber with buffers sparged with nitrogen gas. [Fe–S] cluster reconstitutions of all the metallo-proteins/enzymes were performed in a Coy chamber (97% N_2_, 3% H_2_ atmosphere) with buffers degassed via Schlenk line.

Previously described protocols were followed for the expression and purification of HisTag *Escherichia coli* (*Ec*) SHMT,^32^ HisTag *Ec* T-protein,^32^ and Mb^H64L^,^56^ without modifications; HisTag *Ec* NfuA,^66^ HisTag *Cr*HydA1,^32^ StrepTag WT, and mutant *Ca*HydF^46^ with modifications described below.

### Protein Expression, Purification, and Reconstitution

#### *Ca*HydF with StrepTag

Chemically competent BL21­(DE3) cells were transformed with the pRSF-Duet vector containing the *hydF* gene with an N-terminal StrepTag sequence. A single colony was selected from the fresh transformation plate to start a preculture in phosphate-buffered LB media with 50 μg mL^–1^ kanamycin. Preculture was incubated at 37 °C with 180 rpm shaking overnight. The next day, the seed culture was diluted in 1.5 L of PBS-LB media with 5 g L^–1^ d-glucose and 50 μg mL^–1^ kanamycin, and the culture was incubated at 37 °C with 230 rpm shaking. Overexpression was induced with the addition of 1 mM IPTG when the cell density reached an OD_600_ of 0.6. At the point of induction, cultures were also supplemented with 150 μM ferrous ammonium sulfate (FAS) and incubated at 37 °C with 230 rpm shaking. After 2.5 h of induction, a second aliquot of FAS was added (final 300 μM), and the culture flasks were sparged with nitrogen at 4 °C overnight. Cells were harvested by centrifuging at 9,180 *g*, 4 °C for 10 min. Wet cell pellet was flash frozen in liquid nitrogen and stored at −80 °C. Thirty g frozen cell paste was lysed in 60 mL of 100 mM Tris-HCl, pH 8.0, 250 mM KCl, 5% glycerol buffer (buffer A) with 2 mg mL^–1^ lysozyme, 0.1 mg mL^–1^ DNase, 1% (*v/v*) Triton X-100, 2 mM MgCl_2_, and 2 Pierce protease inhibitor tablets. Lysate was homogenized with an 18G needle and a syringe. After 60 min of incubation with continuous stirring, the lysate was clarified by centrifuging at 4 °C and 11,000*g* for 45 min. Clarified lysate was filtered with 0.22 μm and loaded onto a 5 mL StrepTactin column equilibrated with buffer A. After the column was washed with buffer A, the target protein was eluted with buffer A with 50 mM D-biotin. As-isolated StrepTag *Ca*HydF WT was reconstituted in buffer A with 5 mM DTT following a previously published protocol^67^ by using ferric ammonium citrate as the iron source and lithium sulfide as the sulfur source. After 3 h of incubation, the reconstitution mixture was centrifuged at 11,000*g* for 15 min and then desalted via a 100 mL custom-packed desalting column with G25 resin. Desalted protein stock was concentrated with Amicon Ultra-15 centrifugal filters, flash frozen in liquid nitrogen, and stored at −80 °C. The resulting protein contained 4.25 ± 0.30 Fe/monomer.

#### *Cr*HydA with HisTag

BL21­(DE3) cells were transformed with pETDuet-1-hydA1 with an N-terminal 6x-HisTag construct. Preculturing and overexpression followed the protocol detailed above for *Ca*HydF without changes. Cells were lysed in 50 mM Tris-HCl, pH 8.0, 100 mM KCl, and 5% glycerol buffer with the same lysis components indicated for *Ca*HydF. Lysate was pH adjusted with KOH to pH 8.0. Clarified lysate was loaded onto a 5 mL HisTrap Ni-NTA column equilibrated with 50 mM Tris-HCl, pH 8.0, 100 mM KCl, 5% glycerol, and 10 mM imidazole. A step gradient was applied with increasing concentration of imidazole in the buffer, starting with 10 mM imidazole up to 500 mM imidazole. The protein eluted with a 150 mM imidazole wash. Pure fractions, screened with SDS PAGE, were pooled and desalted via a G25 resin-packed desalting column. As-purified protein was reconstituted and processed as described above for *C.a.* HydF, providing protein with 3.9 ± 0.2 Fe/monomer.

#### *Ec*NfuA with HisTag

Chemically competent BL21­(DE3) cells were transformed with the pET15b-nfuA construct and streaked onto LB-agar plates supplemented with 100 μg mL^–1^ ampicillin. A 10 mL preculture was started in ampicillin-supplemented LB media from a single colony and incubated at 37 °C with shaking overnight. The next day, 3.5 mL of preculture was inoculated into each 2.8 L Fernbach flask containing phosphate-buffered LB media with 100 μg mL^–1^ ampicillin and 5 g L^–1^ d-glucose. Cultures were incubated at 37 °C with 180 rpm shaking. When the cell density reached OD_600_ ≈ 0.5, culture flasks were placed into an ice–water bath for 45 min, followed by induction with freshly prepared 1 mM IPTG. At the time of induction, 0.05 mM ferric ammonium citrate was also added to each culture and incubated at 18 °C with 180 rpm shaking overnight. Cultures were centrifuged the next day at 4 °C, 7,000 rpm for 15 min, and the resulting wet cell paste was flash frozen in liquid nitrogen and stored at −80 °C until further use. Cells were lysed in 50 mM HEPES at pH 7.5, 300 mM KCl, 10% glycerol buffer containing 0.2 mg mL^–1^ lysozyme, 0.01 mg mL^–1^ DNase, 1% (*v/v*) Triton X-100, 2 mg mL^–1^ MgCl_2_, and an EDTA-free Pierce Protease inhibitor tablet. Lysate was incubated at ambient temperature for a total of 1 h and homogenized with an 18G needle and syringe. Lysate was clarified by centrifuging at 4 °C, 17,000 rpm for 45 min. Lysate supernatant was loaded onto a HisTrap Ni-NTA column equilibrated with 50 mM HEPES pH 7.5, 300 mM KCl, 10 mM imidazole, 10% glycerol. A step gradient was applied with increasing concentrations of imidazole (50, 150, 250, and 500 mM) in the buffer. The resulting eluted brown fractions were combined, concentrated with Amicon Ultra-15 spin filters (10 kDa MWCO), and buffer-exchanged to 50 mM HEPES, pH 7.5, 300 mM KCl, 10 mM imidazole, 20% glycerol via a PD-10 column. Buffer-exchanged protein was concentrated with Amicon Ultra-15 spin filters (10 kDa MWCO), flash frozen with liquid nitrogen, and stored at −80 °C until reconstitution. A previously published protocol was followed for the reconstitution of HisTag *E.c.* NfuA, providing protein with 1.96 ± 0.12 Fe/monomer.^67^

#### *C.a* HydF^C353A, C356A^ with StrepTag

WT strep-tagged *C.a.* HydF construct (pRSF-Duet vector) was used as the template for site-directed mutagenesis by PCR, as described in the SI. The sequence-verified construct was used to transform BL21­(DE3) cells. The protocol described above for the WT HydF was followed for the expression and purification of strep-tagged C353A, C356A double mutant *C.a.* HydF, except that the growth medium was not supplemented with ferric ammonium citrate; culture agitation was kept at 200 rpm, and the lysis mixture contained 2 mg mL^–1^ MgCl_2_.

#### *C.a* HydF^D311C^ with StrepTag

The site-directed mutagenesis protocol described above for HydF^C353A,C356A^ was followed, but with appropriate primers as described in Table S1 in the SI. The expression, purification, and reconstitution of *C.a.* HydF^D311C^ followed the same protocol as described for StrepTag *C.a.* HydF WT, except the lysis mixture was supplemented with 2 mg mL^–1^ MgCl_2_. The resulting protein contained 3.92 ± 0.20 Fe/monomer.

#### Heme Reconstitution of Mb^H64L^

Inside an anaerobic Coy chamber (3% H_2_, 97% N_2_), purified H64L Myoglobin (Mb) was resuspended in 20 mM Tris at pH 8. Dithiothreitol (DTT) was added to a final concentration of 5 mM, and the solution was stirred for 10 min, after which sodium dithionite (NaDT) was added to 5 mM. Ferroprotoporphyrin (Cayman Chemical) solubilized with 0.1 M NaOH and subsequently buffered with 20 mM Tris, pH 8, was added slowly until molar equivalency with H64L Mb was reached. The solution was incubated while stirring for 3 h, and reconstitution was monitored by UV–visible spectroscopy of heme absorption at the Soret absorbance maxima (λ_max_ = 432 nm). After stabilization of the absorption spectrum, the solution was loaded on a PD-10 desalting column with Sephadex G25 resin (Cytiva).

### [2Fe]_E_ Synthesis

The synthesis of [2Fe]_E_ ([Fe_2_(μ-SH)_2_(CN)_2_(CO)_4_]^2–^) was carried out as previously described.^33^ This synthesis was based on that described by Rauchfuss and coworkers,^30^ which has been further elaborated in a recent publication.^68^

### Maturation Reactions

The *in vitro* maturation assays were conducted in an MBraun UNIlab anaerobic chamber (O_2_ < 0.5 ppm) at ambient temperature as previously described with the following modifications. A typical 200 μL maturation mixture contained 4 μM HydA, 40 μM HydF (WT or variant), 10 μM T-protein, 5 μM SHMT, 50 μM NfuA, 200 μM MbH64L, 80 μM [2Fe]_E_, 10 μM pyridoxal phosphate (PLP), 1 mM dithiothreitol (DTT), 2 mM dithionite (DT), 44 mM l-serine and 47 mM NH_4_Cl, 2 mM MgCl_2_, and 20 mM GTP. Maturation mixtures were incubated in the dark, and aliquots were taken at specified time points and assayed for hydrogen production.

### Hydrogenase Activity Assays

Hydrogenase assays contained 10 mM methyl viologen, 20 mM NaDT, and 4 nM HydA in 50 mM Tris-HCl pH 6.9 buffer. Assays were started in a glovebox, and the sealed 25 mL crimp vial was then taken outside and incubated in a water bath at 37 °C. 100 μL headspace aliquots were taken and injected onto a GC to monitor H_2_ production.

### [2Fe]_E_ Binding Assays

In order to track [2Fe]_E_ binding to HydF, Apo, D311C, and C353A,C356A proteins at 50 μM concentration, respectively, were each incubated with a 5-fold excess of [2Fe]_E_ for 15 min in the dark and in the absence of reducing agents within an MBraun UNIlab chamber (O_2_ < 0.1 ppm). Samples were then each subjected to extensive buffer exchange events using Amicon Ultra 0.5 mL, MWCO 30 kDa spin filters and 50 mM HEPES, pH 7.55, 250 mM KCl buffer. Final protein samples were characterized by Bradford and atomic absorption (AA) analyses.

Protein aliquots for AA were diluted into 2.5% nitric acid in 2 mL O-ring capped cryovials. Samples were then heated for 30 min at 95 °C, and then centrifuged for 3 min at 14.8 k rpm. The resulting supernatant was carefully decanted and filtered into clean 2 mL cryovials using a syringe fitted with a 0.22 μm filter that was prerinsed with MQ-H_2_O. The total iron in the HydF samples was quantified via a Varian SpectrAA 220 FS flame atomic absorption spectrometer. A 0.4–2.0 ppm standard curve was utilized by dilution from a 1000 ppm iron AA standard solution (Ricca Chemical Company) into 2.5% nitric acid.

### EPR Sample Preparation

Samples of WT strep-tagged HydF, D311C, and C353A,C356A proteins were characterized by EPR spectroscopy. Sample preparation took place within an MBraun chamber (≤0.5 ppm of O_2_) using freshly degassed buffers. WT HydF (45 μM) was incubated with 1 mM NaDT for 8 min in 50 mM HEPES, pH 8.0, 150 mM KCl, and 10% glycerol buffer. D311C (40 μM) and C353A,C356A (40 μM) proteins were incubated with 2 mM NaDT for 10 min in 50 mM HEPES, pH 7.5, 250 mM KCl, and 5% glycerol buffer. In all cases, enzyme and reducing agent were transferred to EPR tubes (Wilmad LabGlass, 4 mm OD, NJ, USA), capped with rubber septa, and then flash frozen in liquid N_2_ outside the MBraun chamber at the indicated time points. EPR samples were stored in a liquid N_2_ dewar until data acquisition occurred.

A series of samples was generated in order to probe the ability of NfuA to load the [4Fe-4S] cluster in apoHydF. ApoHydF (40 μM) was incubated with NfuA (50 μM dimer) in the presence of 1 mM DTT and 2 mM NaDT. Control samples included ApoHydF (40 μM) in the presence of 1 mM DTT and 2 mM NaDT, as well as NfuA (50 μM dimer) in the presence of 1 mM DTT and 2 mM NaDT. Samples were prepared within an MBraun chamber (≤0.1 ppm of O_2_) using 50 mM HEPES, pH 7.55, 250 mM KCl buffer. Samples were transferred to EPR tubes (Wilmad LabGlass, 4 mm OD, NJ, USA), capped with rubber septa, and then flash frozen in liquid N_2_ outside the MBraun chamber immediately following a 1 h incubation period at ambient temperature. Samples were stored in a liquid N_2_ dewar until data acquisition occurred.

### EPR Spectroscopy

A Bruker EMX spectrometer was used to collect continuous wave (CW), X-band (9.38 GHz) spectra. The instrument is equipped with a ColdEdge (Sumitomo Cryogenics) 10 K waveguide in-cavity cryogen-free system attached to a helium Stinger recirculating unit (Sumitomo Cryogenics, ColdEdge Technologies, Allentown, PA); helium gas flow was maintained at 100 psi. A Mercury iTC unit (Oxford Instruments) was used to control the temperature. Spectral parameters for data collection were as follows: 12.0 K, 1.0 mW microwave power, 10 G modulation amplitude, and 100 kHz modulation frequency, and spectra were averaged over 6 scans. Background cavity signals were collected under spectrometer settings identical to those of the HydF samples. HydF spectra were baseline and cavity corrected using OriginPro (2019b, OriginLab Corp., Northampton, MA, USA).

## Supplementary Material



## Abbreviations

DTMA: dithiomethylamine
SAM: *S*-adenosyl-l-methionine
H_met_: aminomethyl-lipoyl-H-protein
SHMT: serine hydroxymethyltransferase
FS: ferrous sulfate
DTT: dithiothreitol
NaDT: sodium dithionite
