# Supplementary material for: The [4Fe-4S] Cluster of HydF Is Essential for [FeFe]-Hydrogenase Maturation
Source: J Am Chem Soc. 2025 Dec 10;147(51):47773–82. doi: 10.1021/jacs.5c18286 (PMC12752695; doi:10.1021/jacs.5c18286)
Supplement: Supplementary file 1 [file ja5c18286_si_001.pdf]

Supporting Information

The [4Fe-4S] Cluster of HydF is Essential for [FeFe]-Hydrogenase Maturation

Batuhan Balci, Eric M. Shepard, Alexander Marlott, Roark D. O'Neill, Michael T. Mock, William E. Broderick, and Joan B. Broderick*

Department of Chemistry & Biochemistry, Montana State University, Bozeman, MT. 59717

Supplemental Methods

Preparation of Apo *Ca*HydF. Overexpression of *Clostridium acetobutylicum (Ca)* HydF was carried out using an *E. coli* BL21(DE3) (Stratagene) cell line containing a construct comprised of 6x-histidine tagged *Ca hydF* (pRSFDuet vector), along with untagged *Ca hydE* (pETDuet vector) and untagged *Ca hydG* (pCDFDuet vector), as described previously.[1] Single colonies from a fresh agar plate supplemented with 30 μg/mL kanamycin, 50 μg/mL ampicillin, and 50 μg/mL streptomycin were chosen for small scale overnight cultures. The following morning, the seed cultures were used to inoculate six 1.5 L flasks (9 L total, 8 mL seed culture per flask) of terrific broth media containing 12 g/L tryptone, 24 g/L yeast extract, 2.2 grams/L KH_2PO_4, 9.4 grams/L K_2HPO_4, 30 μg/mL kanamycin, 50 μg/mL ampicillin, and 50 μg/mL streptomycin. Cultures were grown at 37 °C and 230 rpm shaking until OD_{600} values of ~0.45 were achieved. At this time, flasks were removed from the 37 °C incubator and supplemented with IPTG (1 mM final), ferrous ammonium citrate (4 mM final), sterile filtered glucose (0.5% final (v/v)), L-cysteine (2 mM final), and sodium fumarate (10 mM final). Cultures were then incubated at 30 °C without shaking under an active N_2(g) sparge. After a period of 20 hrs, cells were harvested by centrifugation and cell pellets were then flash frozen in liquid N_2. Cell mass was recorded and cell pellets were stored at -80 °C until purification.

The purification of histidine tagged *Ca*HydF was carried out under anaerobic conditions in a Coy anaerobic chamber (Grass Lake, MI) maintained with a 97% N_2 (g), 3% H_2 (g) atmosphere. The lysis and subsequent purification of HydF utilized 50 mM HEPES pH 7.4, 0.3 M KCl, 5% glycerol buffers supplemented with imidazole and was carried out as described in [2]. Elution was accomplished via a step-gradient of imidazole using a 5 mL HisTrap™ Ni^{2+}-affinity column (GE Healthcare) attached to an ÄKTA Basic 100 FPLC (GE Healthcare); absorbance was tracked at both 280 nm and 415 nm wavelengths. Purity was judged by SDS-PAGE analysis and demonstrated that all eluted fractions were highly pure HydF (**Figure S2**). Interestingly, during the elution process the absorbance values at 415 nm, which are characteristic of $[4Fe\text{-}4S]^{2+}$ cluster binding, remained at baseline readings. Fractions of pure HydF were pooled together and dialyzed into 50 mM HEPES, pH 7.5, 0.25 M KCl, 5% glycerol buffer. Aliquots of the dialyzed enzyme were flash frozen in liquid N_2 and stored at -80 °C. Concentration was determined via Bradford assay using a bovine serum albumin standard solution (Thermo Scientific).

Simulation of EPR Spectra. Simulations were performed in the EasySpin software within Matlab.[3] Simulation results were exported and plotted using Origin 2019b. **Figure 3C** shows the experimental spectra for WT strep-tagged HydF, D311C, and C353A,C356A proteins (black) at 12 K and the corresponding simulated spectra for WT and D311C (red) data sets. Simulations for WT enzyme yielded an axial $[4Fe\text{-}4S]^{+}$ cluster signal with g-values of 2.058, 1.877, and 1.863 with corresponding g-strain values of 0.037, 0.037, and 0.073. Simulations for D311C enzyme yielded an axial $[4Fe\text{-}4S]^{+}$ cluster signal with g-values of 2.057, 1.880, and 1.863 with corresponding g-strain values of 0.038, 0.036, and 0.077. The simulated signals are similar to previous assignments made for WT HydF.[4]

Docking $[2Fe]_E$ in HydF. The binding of $[2Fe]_E$ to HydF was investigated by molecular docking simulations using open-source docking software AutoDock Vina (v1.2.7).[5] The [4Fe-4S] cluster of monomeric *Thermosipho melanesiensis (T.me.)* HydF (5KH0) was removed in Pymol prior to processing. The $[2Fe]_E$ ligand file was prepared by first creating a .xyz file using the published computationally derived coordinates for the axial-axial (aa) isomer of $[2Fe]_E$.[6] These input files were processed using AutoDock Tools. Polar hydrogens were added, and water molecules were removed. Docking was restrained to the *T.me.*HydF binding pocket by orienting the grid box to x-center = -16.96, y-center = -

16.284, z-center = -29.45. The grid box dimensions were as follows: x-dimension = 12, y-dimension = 18, z-dimension = 16 (spacing = 1 Angstrom). Docking calculations were performed under the default parameters for AutoDock Vina.

Supplemental Figures and Tables

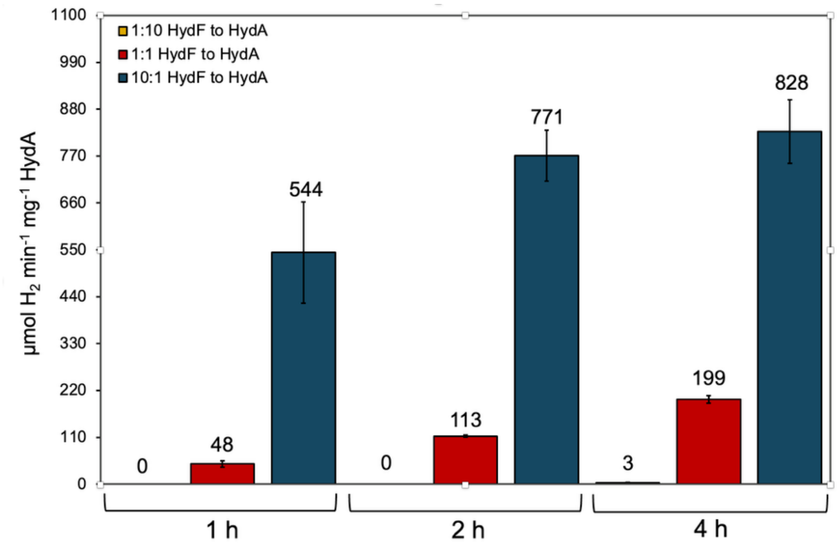

Figure S1. Semisynthetic maturation of *Cr*HydA using wild type *Ca*HydF and $[2Fe]_E$ with Mb^{H64L} and NfuA. Different ratios of HydF to HydA were used, as indicated.

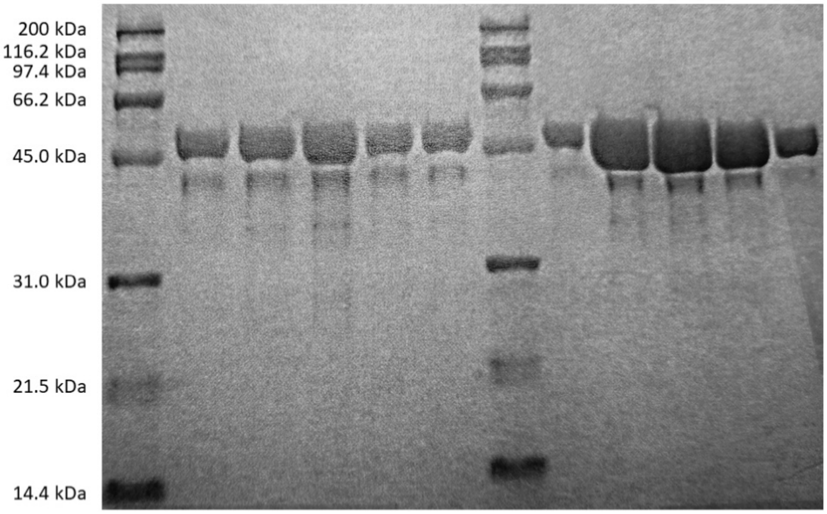

Figure S2. 10% SDS-PAGE gel of HydF$^{(HIS)EG}$ purification fractions eluting at 20% imidazole (lanes 2 – 6) and 50% imidazole (lanes 8 – 12). Lanes 1 and 7: Biorad Broad Range SDS-Page molecular weight standard. *Ca*HydF(6x-histidine tagged) protein MW is 47.39 kDa.

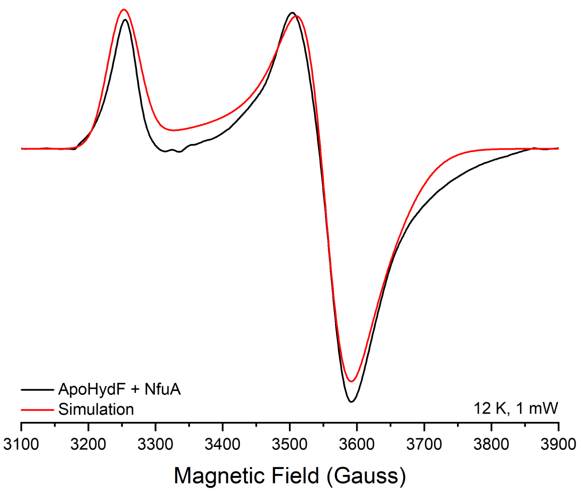

Figure S3. CW X-band EPR spectrum of apo-HydF after incubation with holo-NfuA, reproduced from **Figure 5** (black) overlaid with a simulation (red, g = 2.058, 1.877, and 1.863). EPR parameters: 12.0 K, 1 mW microwave power, 10 G modulation amplitude and 100 kHz modulation frequency

Full Maturation Conditions

	Conc.
Tyrosine	0.4 mM
Cysteine	0.2 mM
Serine	50 mM
NH_4Cl	25 mM
$MgCl_2$	1 mM
PLP	10 uM
DTT	1 mM
CrHydA1	4 uM
TiHydG	25 uM
TmHydE	5 uM
CaHydF-WT or CaHydF-C353A/C356A	5 uM
AMT	10 uM
SHMT	5 uM
NfuA dimer	25 uM
H64L Mb	200 uM
SAM	2.5 mM
DT	2 mM
GTP	20 mM

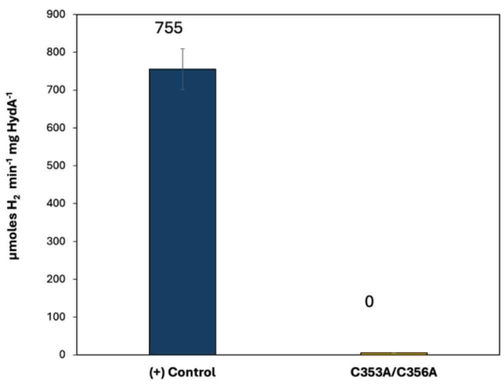

Figure S4. Maturation of *Cr*HydA in the full enzymatic system using either WT HydF or the cluster-knockout variant $HydF^{C353A,C356A}$. Assay components are shown in the table at left.

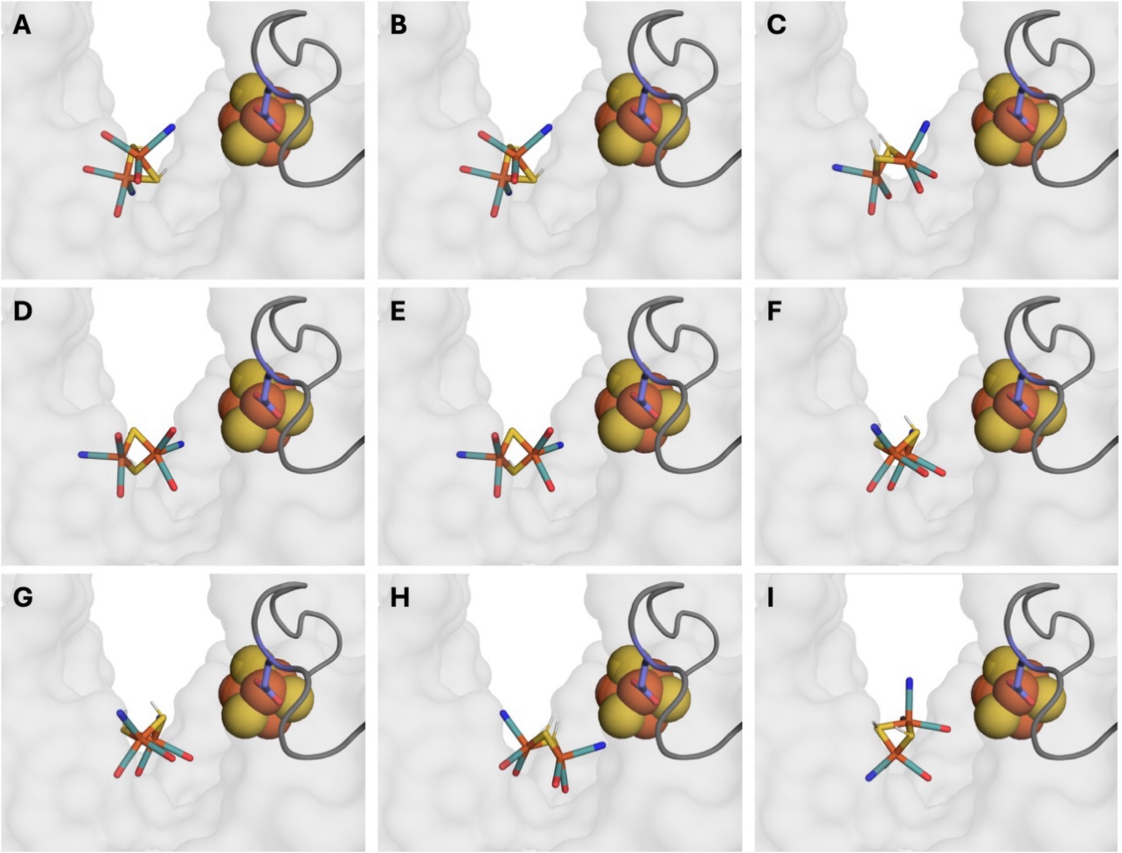

Figure S5. The predicted orientations of $[2Fe]_E$ (aa isomer)[6] in the binding pocket of *T.me.* HydF as calculated by molecular docking simulation software AutoDock Vina. The nine configurations shown are ordered from highest binding affinity for **A** and **B** (-5.9 kcal/mol) to lowest binding affinity for **I** (-5.1 kcal/mol). The docking models show that multiple different binding orientations are possible for $[2Fe]_E$, and as discussed in the main manuscript, the preferred binding mode could be impacted by the presence of the [4Fe-4S] cluster (which was removed during simulations). The highlighted amino acid residue is D311.

Table S1. Primers used in site-directed mutagenesis

Variant	Primer
D311C	Forward: 5′-ccgtcaatcttgcgatataggtaaagtaaaaataccaag-3′
	Reverse: 5′-tggtgggtgcaggcttctg-3′
C353A,C356A	Forward: 5′-atagttcatgctgctggcgctatgctaaacagacgttcaatg-3′
	Reverse: 5′-aagtgcataatcctctatatttggaggaaatgaaaagccact-3′

Supplemental References

(1) McGlynn, S. E.; Shepard, E. M.; Winslow, M. A.; Naumov, A. V.; Duschene, K. S.; Posewitz, M. C.; Broderick, W. E.; Broderick, J. B.; Peters, J. W. HydF as a scaffold protein in [FeFe] hydrogenase H-cluster biosynthesis. *FEBS Lett.* **2008**, *582* (15), 2183-2187.

(2) Shepard, E. M.; Byer, A. S.; Betz, J. N.; Peters, J. W.; Broderick, J. B. A Redox Active [2Fe-2S] Cluster on the Hydrogenase Maturase HydF. *Biochemistry* **2016**, *55* (25), 3514-3527. DOI: 10.1021/acs.biochem.6b00528.

(3) Stoll, S.; Schweiger, A. EasySpin, a comprehensive software package for spectral simulation and analysis in EPR. *J. Magn. Reson.* **2006**, *178* (1), 42-55.

(4) Scott, A. G.; Szilagyi, R. K.; Mulder, D. W.; Ratzloff, M. W.; Byer, A. S.; King, P. W.; Broderick, W. E.; Shepard, E. M.; Broderick, J. B. Compositional and structural insights into the nature of the H-cluster precursor on HydF. *Dalton Trans.* **2018**, *47* (28), 9521-9535. DOI: 10.1039/c8dt01654b.

(5) Trott, O.; Olson, A. J. AutoDock Vina: Improving the speed and accuracy of docking with a new scoring function, efficient optimization, and multithreading. *J. Comp. Chem.* **2010**, *31* (2), 455-461.

(6) Zhang, Y.; Tao, L.; Woods, T. J.; Britt, R. D.; Rauchfuss, T. B. Organometallic $Fe_2(u\text{-}SH)_2(CO)_4(CN)_2$ cluster allows the biosynthesis of the [FeFe]-hydrogenase with only the HydF maturase. *J. Am. Chem. Soc.* **2022**, *144* (4), 1534-1538.
